# Correction to: ‘A polycarboxylic/ether composite polymer electrolyte via *in situ* UV-curing for all-solid-state lithium battery’ (2020) by Wang *et al.*

**DOI:** 10.1098/rsos.262352

**Published:** 2026-10-28

**Authors:** Wenli Wang, Ziwen Qiu, Qian Wang, Xiaoyu Zhou, Yang Liu, Jingjing Zhou, Bingkun Guo

**Affiliations:** ^1^ Materials Genome Institute, Shanghai University, Shanghai, 200444, People's Republic of China; ^2^ Guangdong Provincial Key Laboratory of Energy Materials for Electric Power, Southern University of Science and Technology, Shenzhen, 518055, People's Republic of China

**Keywords:** composite polymer electrolyte, *in situ*, matrix regulating, all-solid-state, battery

The following text corrects the paper: Wang *et al.* 2020 A polycarboxylic/ether composite polymer electrolyte via *in situ* UV-curing for all-solid-state lithium battery. *R. Soc. Open Sci.* **7**: 200598. https://doi.org/10.1098/rsos.200598.

The primary purpose of figure 1b in this work is to intuitively illustrate the glass transition temperatures of the three selected materials, enabling straightforward visual identification of the key glass transition feature for each sample. To eliminate random high-frequency noise in the raw poly(ethylene oxide) (PEO) dataset that could obscure the critical inflection point corresponding to the glass transition stage, a smoothing operation (10–50 pts) was exclusively applied to the PEO data series in the visualization of figure 1b. This operation does not affect the rigour and authenticity of all quantitative results and performance comparison conclusions presented in the paper.

To clarify this, the figure 1 caption should read: ‘FTIR (a) and DSC (b) curves of the SSE films. A smoothing operation (10–50 pts) was applied solely to the PEO DSC curve for visualization to reduce high-frequency noise’.

