# Supplementary material for: A polycarboxylic/ether composite polymer electrolyte via in situ UV-curing for all-solid-state lithium battery
Source: R Soc Open Sci. 2020 Jul 15;7(7):200598. doi: 10.1098/rsos.200598 (PMC7428264; doi:10.1098/rsos.200598)

Supporting Information

A polycarboxylic/ether composite polymer electrolyte via in situ UV-curing for all-solid-state lithium battery

1. Experimental Section.
2. Figure S1. The XRD patterns of the composites with different content of PEO.
3. Experimental Section

LiFePO_4_, acetone black, aluminum (Al) foil and polyvinylidene fluoride (PVDF) were purchased from Hefei Kejing Materials Technology CO., Ltd, China. Polyethylene oxide (PEO), lithium bistrifluoromethanesulfonimide (LiTFSI), di(ethylene glycol) diacrylate (A2, 98 %), 2-hydroxy-2-methyl-1-phenyl-1-propanone (HMPP, 97 %) and the solvents were bought from Alfa Asear, stored in an argon-filled glovebox (H_2_O < 1 ppm, O_2_ < 1 ppm) and used without further purification.

The cathodes and SSEs were prepared as the literature reported.^[22]^ The vacuum dried SSE films were measured on a fourier-transform infrared spectroscopy (FTIR, Nicolet 6700) with the resolution of 0.02 cm^-1^. Morphologies of the solid state electrolytes (SSEs) were excamined by the scanning electron microscope (SEM, ZEISS, MERLIN Compact). The X-ray diffraction (XRD) patterns of the samples were obtained on a D8 with Cu K radiation. Differential scanning calorimetry (DSC) measurements were carried out from 20 to 120 °C at the heating rate of 10 °C min^-1^ in N_2_ atmosphere (NETZSCH, DSC214). The ionic conductivity measurements were conducted on a Solartron 1470E electrochemical workstation in the frequency range from 100 kHz to 1 Hz with the oscillation potential of 10 mV from 25 to 80 °C.

The electrochemical stabilities of the SSEs were tested by linear sweep voltammetry (LSV) on the stainless steel electrodes with lithium foils as the counter electrodes and reference electrodes at 25 °C. The cyclic and rate performances were tested on a LANHE CT2001A.

1. Figure S1. The XRD patterns of the composites with different content of PEO.


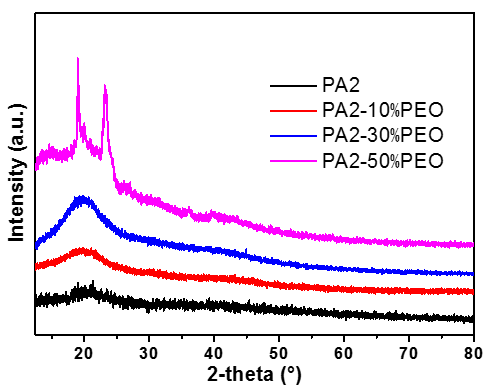


**TOC**


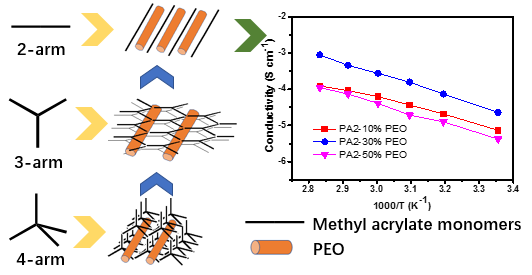

Supplement: Supporting Information [file rsos200598supp1.docx]
