# Supplementary figures and images for: A polycarboxylic/ether composite polymer electrolyte via in situ UV-curing for all-solid-state lithium battery
Source: R Soc Open Sci. 2020 Jul 15;7(7):200598. doi: 10.1098/rsos.200598 (PMC7428264; doi:10.1098/rsos.200598)

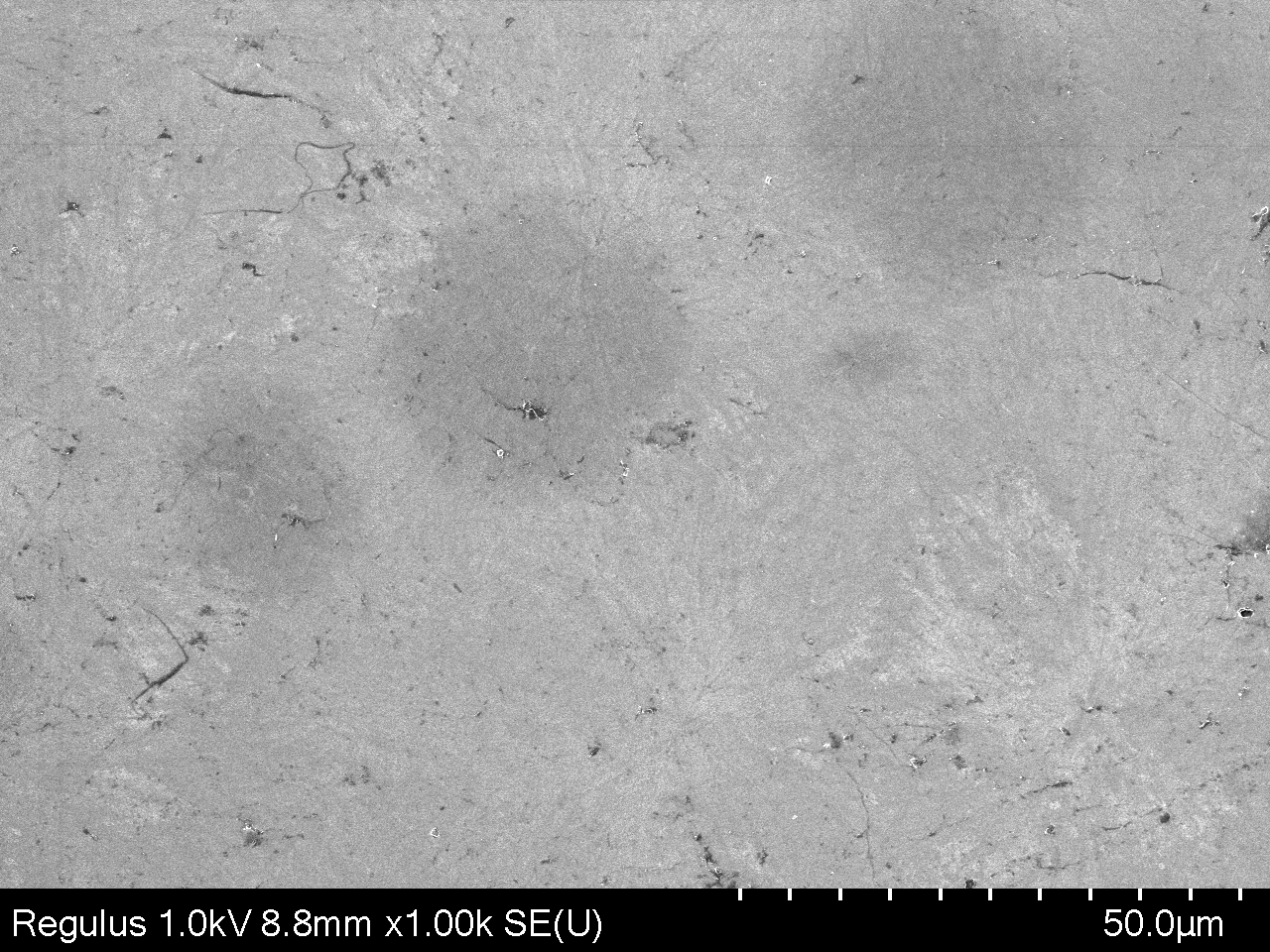

Supplement: Fig.3a.xlsx [file rsos200598supp5.tif]
